# Longitudinal changes of regional spontaneous brain activity in nasopharyngeal carcinoma patients receiving chemoradiotherapy

**DOI:** 10.3389/fnins.2025.1607727

**Published:** 2025-07-25

**Authors:** Jing Wu, Huanfeng Zhu, Siwen Liu, Li Yin, Jianfeng Wu, Chengyun Yao, Wenjie Guo, Xia He, Na Yin

**Affiliations:** ^1^Department of Radiotherapy, The Affiliated Cancer Hospital of Nanjing Medical University, Jiangsu Cancer Hospital, Jiangsu Institute of Cancer Research, Nanjing, China; ^2^Department of Oncology, The Affiliated Cancer Hospital of Nanjing Medical University, Jiangsu Cancer Hospital, Jiangsu Institute of Cancer Research, Nanjing, China; ^3^Department of Radiology, The Affiliated Cancer Hospital of Nanjing Medical University, Jiangsu Cancer Hospital, Jiangsu Institute of Cancer Research, Nanjing, China

**Keywords:** nasopharyngeal carcinoma, chemotherapy, radiotherapy, resting-state functional magnetic resonance imaging, spontaneous brain activity, default mode network

## Abstract

**Introduction:**

Nasopharyngeal carcinoma (NPC) is a common malignant tumor primarily treated by radiotherapy with or without chemotherapy. Chemoradiotherapy frequently contributes to cognitive impairments, which are associated with abnormal brain activity. This study aimed to longitudinally explore the stage-specific changes of regional spontaneous brain activity in NPC patients during different phases of chemoradiation treatment.

**Methods:**

Twenty patients diagnosed with stage III-IV NPC were enrolled in this study from January 2022 to December 2023. All patients received two cycles of chemotherapy (1st follow-up) followed by one cycle of chemotherapy plus radiotherapy (2nd follow-up). Resting-state functional magnetic resonance imaging (rs-fMRI) data were acquired from all patients at baseline, 1st follow-up and 2nd follow-up. Based on rs-fMRI data after preprocessing, the metrics of regional homogeneity (ReHo) and fractional aptitude of low-frequency fluctuation (fALFF) values were calculated and compared to measure the changes of regional spontaneous activity in the brain.

**Results:**

The NPC patient group showed increased ReHo values in the right middle cingulate gyrus at the 1st follow-up when compared with baseline. In addition, the NPC patient group exhibited increased ReHo values in the left calcarine fissure at the 2nd follow-up when compared with the 1st follow-up. The NPC patient group demonstrated decreased fALFF values in the right inferior temporal gyrus at the 2nd follow-up when compared with baseline.

**Conclusion:**

This longitudinal study revealed distinct stage-specific brain activity changes during chemoradiotherapy in NPC patients. Chemotherapy induced transient compensatory increases in ReHo in the middle cingulate gyrus, while subsequent radiotherapy led to increased activity in the calcarine fissure. Combined treatment resulted in decreased spontaneous activity in the inferior temporal gyrus, a key component of the default mode network. These temporal dynamics suggest evolving compensatory mechanisms followed by eventual functional alterations, providing neurobiological insights into the progressive nature of treatment-related cognitive impairments and potential biomarkers for monitoring brain changes during cancer treatment.

## 1 Introduction

Nasopharyngeal carcinoma (NPC) is a common type of malignant tumor in head and neck, which is highly prevalent in certain geographical areas, such as southern China (Chang et al., 2021). According to Global Cancer Statistics, approximately 133,354 new cases of NPC were diagnosed worldwide in 2020, among which more than 70% were found in in East and Southeast Asia (Sung et al., 2021). The non-keratinizing subtype is a major pathological classification of NPC, which is associated with Epstein–Barr virus infection and accounts for >95% of cases in epidemic areas (Tang et al., 2021). Since NPC is a highly radiotherapy- and chemotherapy-sensitive cancer, single radiotherapy or combined chemotherapy and radiotherapy are the primary treatments for patients with early and locally advanced NPC accounting for 70% of those diagnosed for the first time (Jiromaru et al., 2022). The addition of concomitant chemotherapy to radiotherapy significantly improved the overall survival (85–90% for 5-year) of patients with locally advanced NPC (Blanchard et al., 2015; Perri et al., 2013). However, these treatments are often accompanied by side effects that seriously affect the prognosis and quality of life of survivors (Tang et al., 2012). Neurological complications, especially cognitive impairments, have been reported in NPC patients following radiotherapy or/and chemotherapy (Ma et al., 2016; Zhang et al., 2021; Cheung et al., 2003). Radiation-induced brain abnormalities were dose-dependent and volume-dependent in NPC patients following radiotherapy, which suggested that neuroimaging methods might provide early biomarkers for cognitive impairments of NPC patients receiving radiotherapy (Voon et al., 2023). In addition, radiation encephalopathy could be facilitated by chemotherapeutic agents associated- structural and functional brain alterations in NPC patients who underwent radiotherapy (Zhang et al., 2019). The cognitive impairments including abnormal attention, memory and executive functions, as well as functional deficits such as speech, hearing and swallowing, were related to brain functional changes (prefrontal, cingulate, parietal, temporal, occipital cortex and hippocampus, etc.) induced by chemoradiotherapy (Deprez et al., 2013; Yao et al., 2023; Feng et al., 2019; Morrison et al., 2022; Yang et al., 2019).

Therefore, it is essential to understand the effects of radiotherapy or/and chemotherapy on the activity of brain regions, especially those involved in the cognitive processes. The technique of resting-state functional magnetic resonance imaging (rs-fMRI) has been widely used for measuring the changes of local spontaneous activity in the brain. The regional homogeneity (ReHo) and fractional aptitude of low-frequency fluctuation (fALFF) are the two major rs-fMRI metrics to address regional brain alterations (Zou et al., 2008; Liu et al., 2010). Carboplatin-based chemotherapy could cause cognitive impairments, which might be related to the changes of activity in the prefrontal cortex, insula and caudate measured by ReHo values (Liu et al., 2022). Decreased fALFF values in the left anterior cingulate gyrus, middle frontal gyrus and increased fALFF values in the left superior frontal gyrus (orbital part), middle occipital gyrus had been detected in colorectal cancer patients after chemotherapy, which were associated with cognitive impairments of patients (Liu et al., 2022). In a previous rs-fMRI study, radiotherapy-induced brain functional alterations were also revealed in NPC patients, which might serve as the potential biomarkers for radiotherapy-induced cognitive impairments and provide valuable targets for further functional recovery treatments (Ma et al., 2016). From the perspective of functional networks, reduced network efficiency and reduced functional connectivity were discovered in patients after NPC radiotherapy using rs-fMRI and graph theoretical analysis (Leng et al., 2021). However, previous research has mainly focused on exploring the effects of radiotherapy on the brain in NPC patients. Little is known about the longitudinal changes of brain activity in NPC patients receiving chemoradiotherapy.

In this study, we speculated that NPC patients might exhibit differences in the local spontaneous brain activity at different stages of chemotherapy plus radiotherapy treatment. In order to verify this hypothesis, the metrics of ReHo and fALFF values were calculated and compared to identify brain regions with altered activity based on preprocessed rs-fMRI data.

## 2 Materials and methods

### 2.1 Participants

The current study was approved by the Ethical Commission of Jiangsu Cancer Hospital and Jiangsu Institute of Cancer Research and The Affiliated Cancer Hospital of Nanjing Medical University. Each patient signed informed consent before participation. In this study, NPC was diagnosed by nasopharyngeal biopsy and histopathology according to the 8th edition of Head and Neck Tumor Staging Criteria for NPC (Huang and O'sullivan, 2017). Twenty pathologically confirmed non-cornification undifferentiated NPC patients (T_3-4_N_0-1_M_0_ or T_1-4_N_2-3_M_0_) were included in this study from January 2022 to December 2023. According to the National Comprehensive Cancer Network (NCCN) guidelines for NPC (Zhou et al., 2020), all patients received two cycles of paclitaxel combining with platinum chemotherapy (1st follow-up). After two cycles of chemotherapy, all patients underwent one cycle of chemotherapy (paclitaxel combining with platinum) plus radiotherapy (2nd follow-up). They were irradiated with 28–33 fractions (once a day, quintic a week). The prescribed radiation dose to gross tumor volume of the nasopharynx (GTVnx) was 70–72 Gy, to gross tumor volume of lymph nodes (GTVnd) was 66–70 Gy, to high-risk clinical target volume (CTV1) was 60 Gy, and to low-risk clinical target volume (CTV2) was 50.4 Gy.

The inclusion criteria were as follows: (1) Han Chinese ethnicity, right-handed, aged from 20 to 60 years old and more than 9 years of education; (2) pathologically confirmed non-cornification undifferentiated carcinoma (T_3-4_N_0-1_M_0_ or T_1-4_N_2-3_M_0_); (3) general good condition (KPS) ≥ 70 or Eastern Cooperative Oncology Group (ECOC): 0–1; (4) without severe concurrent medical or surgical diseases, and no concurrent malignancies; (5) all the patients were initial treatment who never received any chemotherapy or radiotherapy; (6) normal-appearing brain parenchyma on MRI.

The exclusion criteria were as follows: (1) combined with other malignant tumors or distant metastasis (M1); (2) incomplete clinical information or pathological information; (3) current or history of chemotherapy or radiotherapy; (4) current or history of any neurological, psychiatric disorders or significant brain injury (i.e., structural abnormalities on conventional MRI); (5) any history of alcohol or drug dependence; (6) any contraindication to MRI scanning.

In this study, 20 patients (age: 48.0 ± 9.4; 14 male and 6 female) histologically diagnosed of non-cornification undifferentiated NPC were enrolled. There were 4 patients in T1, 1 patient in T2, 11 patients in T3, 4 patients in T4. There were 3 patients in N0, 6 patients in N1, 9 patients in N2, 2 patients in N3. There were 20 patients in M0 and no patient in M1. According to the staging criteria of American Joint Committee on Cancer (AJCC), there were no patient in stage I and II, 14 patients in stage III, 6 patients in stage IVA based on the T-, N-, and M-classification results. The demographic and clinical characteristics of patients were presented in Table 1.

**Table 1:** Demographic and clinical characters of patients.

Characteristics	NPC (*n* = 20)
Age (years)	48.0 ± 9.4
Gender (male/female)	14/6
Handedness (right/left)	20/0
Pathological classification (*n*)	
Non-cornification undifferentiated	20
Non-cornification differentiated	0
T-classification (*n*)	
T1	4
T2	1
T3	11
T4	4
N-classification (*n*)	
N0	3
N1	6
N2	9
N3	2
M-classification (*n*)	
M0	20
M1	0
AJCC TMN stage (*n*)	
I	0
II	0
III	14
IVA	6
Chemotherapy regimen	
Paclitaxel combining with platinum (*n*)	20
Radiotherapy regimen (Gy)	
GTVnx	70–72
GTVnd	66–70
CTV1	60
CTV2	50.4

NPC, nasopharyngeal carcinoma; T-classification described the size of the primary tumor and whether it had invaded nearby tissue; N-classification described nearby lymph nodes that were involved; M-classification defined distant metastasis; AJCC, American Joint Committee on Cancer; AJCC TMN stage was obtained based on the T-, N-, and M-classification results; GTVnx, gross tumor volume of the nasopharynx; GTVnd, gross tumor volume of lymph nodes; CTV1, high-risk clinical target volume; CTV2, low-risk clinical target volume.

### 2.2 MRI data acquisition, preprocessing and metrics calculation

**Figure 1:**
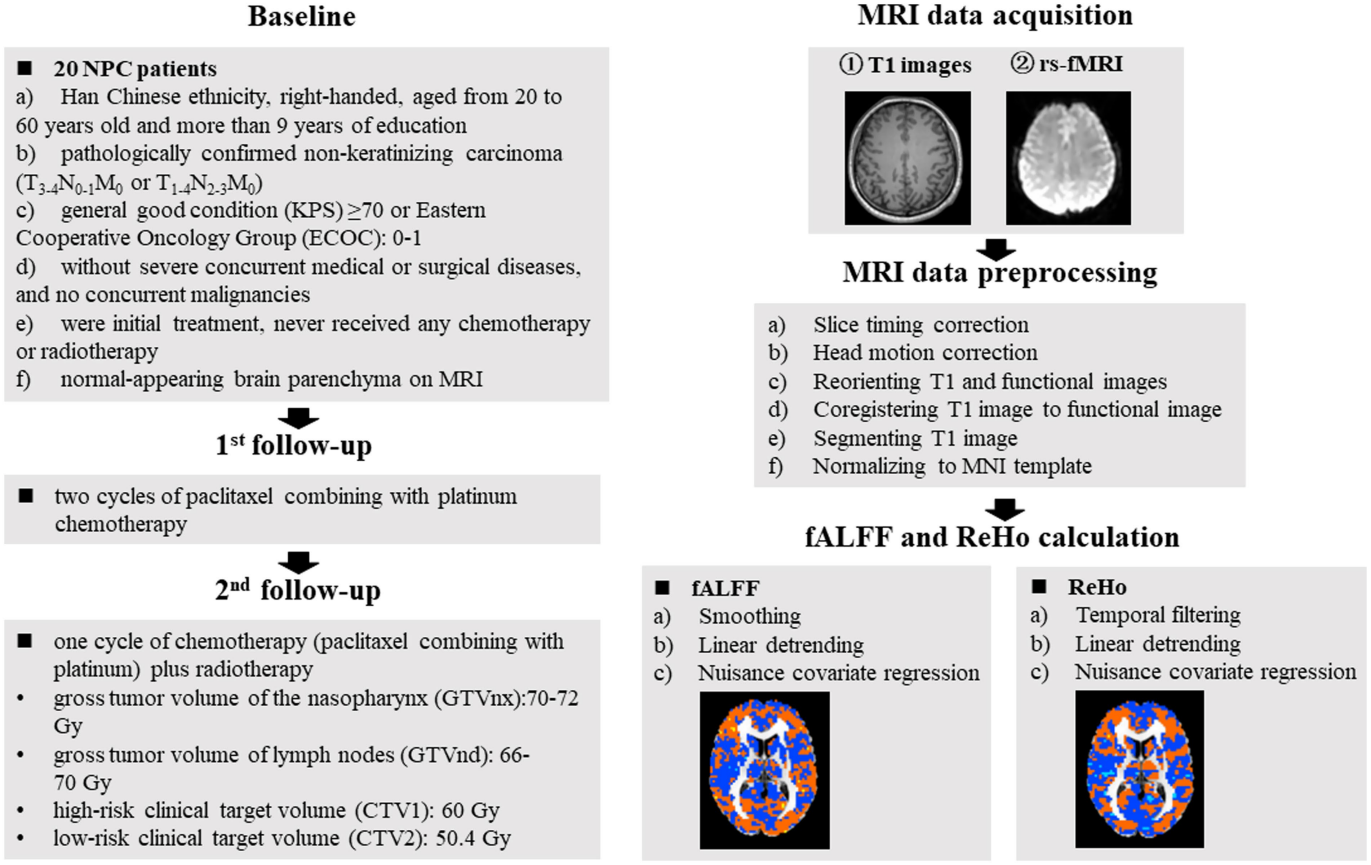
Schematic overview of the study. The red dots in the brain represented increased brain activity; the blue dots in the brain represented decreased brain activity. NPC, nasopharyngeal carcinoma; fALFF, fractional aptitude of low-frequency fluctuation; ReHo, regional homogeneity.

MRI data were acquired with a 3.0 T Philips Aachieva Scanner at the Department of Radiotherapy, Jiangsu Cancer Hospital and Jiangsu Institute of Cancer Research and The Affiliated Cancer Hospital of Nanjing Medical University. The parameters of T1-weighted and rs-fMRI images acquisition were described in our previous study (Liu et al., 2022). MRI data were preprocessed using the software of DPARSF (Chao-Gan and Yu-Feng, 2010) and the steps were presented in our previous study (Liu et al., 2022). Based on the preprocessed MRI data, the metrics of ReHo and fALFF values were calculated, and the detailed steps were described in our previous studies (Liu et al., 2022; Liu et al., 2022). The detailed procedures of MRI acquisition, preprocessing and metrics calculation were presented in Figure 1.

### 2.3 Statistical analysis

The measures of ReHo and fALFF were compared between patients at baseline, 1st follow-up and 2nd follow-up by paired *t*-tests using the software of Resting-State fMRI Data Analysis (REST) Toolkit based on MATLAB (Song et al., 2011). The statistical threshold was set at *p* < 0.005 using AlphaSim correction in the REST software (cluster size >13 voxels and 10 voxels). To present other relevant brain regions that were analyzed but did not reach statistical significance, two different cutoff values for cluster sizes were used, which would provide a more comprehensive overview of the examined brain regions and their potential roles in the observed effects.

## 3 Results

### 3.1 Changes of ReHo and fALFF values at different stages of treatment

**Figure 2:**
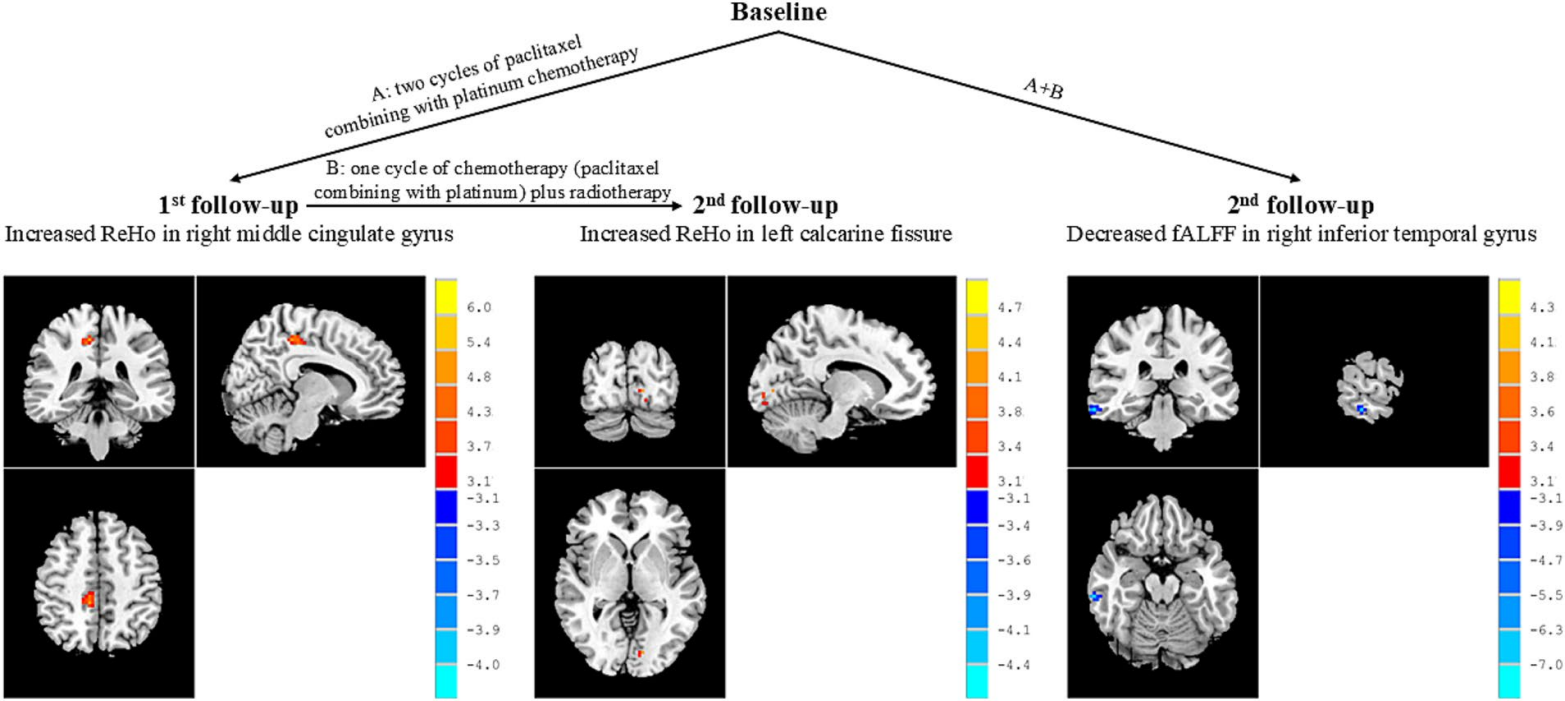
Brain regions showed altered activity between patients at different stages of treatment. Peak *T* values were obtained by paired *t*-tests and corrected by the AlphaSim program in the REST software (*p* < 0.005, cluster size >13 voxels). The color bar on the right represented the size of *T* values. The red dots in the brain represented increased brain activity; the blue dots in the brain represented decreased brain activity. ReHo, regional homogeneity; fALFF, fractional aptitude of low-frequency fluctuation.

The NPC patient group showed increased ReHo values in the right middle cingulate gyrus at the 1st follow-up when compared with baseline (Table 2; Figure 2). There were no significant differences in fALFF values between the NPC patient group at 1st follow-up and baseline.

**Table 2:** Brain regions showed altered activity between patients at baseline, 1st follow-up and 2nd follow-up.

Comparison of brain regions		Peak MNI coordinates			Clusters	Peak *T* values
		x	y	z		
1st follow-up *vs* baseline	Increased ReHo in right middle cingulate gyrus	9	−36	48	33	5.2^a^
	Increased ReHo in right superior frontal gyrus (dorsolateral)	21	21	39	10	5.2^b^
	Decreased ReHo in left middle temporal gyrus	−51	−15	−15	11	−3.9^b^
2nd vs 1st follow-up	Increased ReHo in left calcarine fissure	−12	−84	0	24	4.3^a^
	Increased ReHo in right lingual gyrus	−18	−90	−3	11	4.9^b^
	Increased fALFF in left inferior frontal gyrus (opercular part)	−45	15	9	10	5.0^b^
2nd follow-up vs baseline	Decreased fALFF in right inferior temporal gyrus	66	−30	−18	14	−7.9^a^

^a^Peak T values were obtained by paired *t*-tests and corrected by the AlphaSim program in the REST software (*p* < 0.005, cluster size >13 voxels). ^b^Peak T values were obtained by paired *t*-tests and corrected by the AlphaSim program in the REST software (*p* < 0.005, cluster size >10 voxels). x, y and z were the coordinates of peak voxels of clusters in the MNI space. ReHo, regional homogeneity; fALFF, fractional aptitude of low-frequency fluctuation; MNI, montreal neurological institute.

In addition, the NPC patient group exhibited increased ReHo values in the left calcarine fissure at the 2nd follow-up when compared with 1st follow-up (Table 2; Figure 2). There were no significant differences in fALFF values between patients at 2nd follow-up and 1st follow-up.

The NPC patient group demonstrated decreased fALFF values in the right inferior temporal gyrus at the 2nd follow-up when compared with baseline (Table 2; Figure 2). There were no significant differences in ReHo values between the NPC patient group at 2st follow-up and baseline.

## 4 Discussion

This longitudinal rs-fMRI study revealed distinct patterns of brain activity changes in NPC patients during different stages of chemoradiotherapy. Our findings demonstrate that chemotherapy and radiotherapy induce specific regional alterations in spontaneous brain activity, potentially reflecting compensatory mechanisms and treatment-related neurotoxicity.

### 4.1 Chemotherapy-induced changes in the middle cingulate gyrus

Our study showed increased ReHo values in the right middle cingulate gyrus after two cycles of paclitaxel plus platinum chemotherapy compared to baseline. This finding is consistent with previous functional imaging studies demonstrating chemotherapy-induced hyperactivity in cingulate regions (Omran et al., 2021). Specifically, neurotoxic chemotherapeutic agents have been shown to lead to chronic brain hyperactivity, with platinum-based chemotherapy causing increased functional connectivity in the anterior cingulate cortex and elevated fluorodeoxyglucose uptake in this region. Oxaliplatin chemotherapy has been particularly associated with hyperactivity in the cingulate cortex in both animal models and human studies.

The cingulate cortex, a principal cortical region of the limbic circuitry involved in emotional and cognitive integration, is particularly vulnerable to chemotherapy-induced neurotoxicity. Structurally, cisplatin treatment has been shown to reduce the coherence of white matter fibers and decrease dendritic arborization and spine density within this region. Importantly, these structural abnormalities appear to be reversible, as co-administration of neuroprotective agents such as metformin effectively prevents such damage and restores cognitive function (Zhou et al., 2016; Chiang et al., 2020; Milutinovic et al., 2023).

The temporal pattern of our findings provides crucial insights into treatment-related brain changes. The increased activity in the middle cingulate gyrus was observed only during the first follow-up after chemotherapy, but disappeared during the second follow-up after combined treatment (You et al., 2020). This suggests a dynamic compensatory mechanism whereby the brain initially responds to chemotherapy-induced structural damage with increased functional activity (Andryszak et al., 2017). However, this compensation appears to diminish with continued treatment, potentially leading to subsequent cognitive decline (Li and Caeyenberghs, 2018). This pattern aligns with clinical observations that cognitive impairments often become more pronounced with prolonged treatment.

### 4.2 Radiotherapy-induced changes in the calcarine fissure

The increased ReHo values in the left calcarine fissure observed after chemoradiotherapy (second follow-up) compared to chemotherapy alone (first follow-up) suggests a radiotherapy-specific effect. The calcarine fissure belongs to the primary visual cortex and is crucial for visual processing. Previous studies have shown that chemotherapy can cause smaller gray matter volume in the calcarine fissure, associated with subtle deficits in visual processing and recognition memory (Zong et al., 2025; Oliveira et al., 2023; Einarsson et al., 2018). Additionally, Cognitive impairment has been linked to decreased functional connectivity density in the calcarine fissure (Zhu et al., 2022).

However, our finding of increased regional homogeneity differs from these previous reports of decreased activity or connectivity. This discrepancy may be explained by the additive effects of radiotherapy on chemotherapy-induced changes. Radiotherapy has been shown to cause both increased and decreased ReHo values in brain regions within and outside the radiation field, with increased ReHo potentially associated with the development of radiation encephalopathy (Seitzman et al., 2023; Rübe et al., 2023). Neoadjuvant chemotherapy has previously been associated with increased cerebral blood flow in the bilateral calcarine cortex, and increased perfusion in this region has been related to dysfunction of executive control (Chen et al., 2017).

The specific involvement of visual processing areas in our study may contribute to the visual and cognitive deficits commonly reported in NPC patients following radiotherapy. This finding represents, to our knowledge, the first report of increased consistency of local activity in the left calcarine fissure of NPC patients after chemoradiotherapy, highlighting the need for further experimental validation of the underlying mechanisms.

### 4.3 Combined treatment effects on the inferior temporal gyrus

The decreased fALFF values in the right inferior temporal gyrus observed only after completion of all treatments (compared to baseline) suggests that this change requires the combined and prolonged effects of chemotherapy and radiotherapy. The inferior temporal gyrus is a crucial brain region involved in higher cognitive functions, including semantic memory processing, visual perception, and memory formation (Convit et al., 2000; Chan et al., 2001; Lu et al., 2024; Scheff et al., 2011). Neuronal atrophy in this region has been associated with cognitive dysfunction, including impaired semantic memory processing and sensory integration.

Previous studies have shown differential effects of chemotherapy and radiotherapy on temporal lobe function. Adjuvant chemotherapy can cause cortical thinning and gray matter volume reduction in the inferior temporal gyrus, while also potentially leading to increased activity in this region (Daniel et al., 2022; Zheng et al., 2020). In contrast, radiotherapy typically causes hypometabolic changes in the inferior temporal lobe (Lin et al., 2021). Our finding of decreased activity after combined treatment is consistent with radiotherapy’s predominant effect overriding any compensatory increases that might result from chemotherapy alone.

The inferior temporal gyrus is also a key component of the default mode network (DMN), which plays an important role in self-cognition, emotional processing, and regulation. The DMN has been consistently implicated in chemotherapy-related cognitive impairment, with studies showing reduced resting-state functional connectivity within DMN regions and functional disconnection in the medial temporal lobe associated with attention dysfunction (Zhang et al., 2019). Radiotherapy has similarly been shown to cause reduced functional connectivity within the DMN (Mazio et al., 2022). The preferential vulnerability of the DMN to aging and sensitivity to drug toxicity makes it a potential neuroimaging biomarker of treatment-related brain injury (Kesler, 2014; Chen et al., 2021).

Furthermore, radiotherapy can cause selective and time-dependent white matter atrophy in the right inferior temporal gyrus, which correlates with progressive cognitive deficits (Lin et al., 2021). These alterations are both time-dependent and dose-dependent, suggesting that the magnitude and persistence of changes may be related to treatment intensity and duration.

### 4.4 Clinical implications and temporal dynamics

The differential timing and regional specificity of these changes provide important insights into the pathophysiology of treatment-related cognitive impairment. The sequential appearance of alterations—early cingulate changes after chemotherapy alone, followed by calcarine involvement after radiotherapy, and finally temporal lobe alterations after combined treatment—suggests that different brain regions have varying vulnerabilities to specific treatments and treatment combinations.

This temporal pattern may explain the progressive nature of cognitive decline observed in cancer patients and highlights the importance of longitudinal monitoring throughout treatment. The initial compensatory increases in regional activity (ReHo) in response to both chemotherapy and radiotherapy, followed by eventual decreases in critical cognitive regions (fALFF), suggests a biphasic response pattern that could inform the timing of neuroprotective interventions.

The involvement of regions within established cognitive networks—including the limbic system (cingulate cortex), visual processing areas (calcarine fissure), and the default mode network (inferior temporal gyrus)—provides a neurobiological framework for understanding the multifaceted nature of treatment-related cognitive impairment. These findings suggest that both ReHo and fALFF metrics may serve as sensitive biomarkers for detecting and monitoring treatment-related brain changes, with ReHo potentially more sensitive to early compensatory changes and fALFF more reflective of longer-term functional alterations.

## 5 Limitations

The major limitations of this study include the small sample size (n = 20) and the absence of clinical cognitive assessments before and after treatment. These limitations prevent us from establishing direct correlations between brain activity changes and cognitive performance, which would strengthen the clinical relevance of our neuroimaging findings. Additionally, variations in treatment plans, including differences in chemotherapy drugs, radiation doses, or treatment schedules, might introduce confounding variables that could affect outcomes. Due to the limited sample size, we were unable to analyze dose-dependent effects or stratify patients by different treatment parameters.

The lack of a control group of NPC patients not receiving radiotherapy limits our ability to distinguish treatment-related changes from natural variability over time or disease-related effects. Future studies should include both larger sample sizes and comprehensive cognitive evaluations to validate these findings and establish their clinical significance. Longer follow-up periods would also help determine whether these brain activity changes persist, recover, or continue to evolve after treatment completion.

## 6 Conclusion

This study provides the first longitudinal rs-fMRI evidence of stage-specific brain activity changes in NPC patients receiving chemoradiotherapy. The findings suggest that chemotherapy and radiotherapy induce distinct patterns of regional brain alterations, with initial compensatory increases in regional homogeneity followed by eventual decreases in the magnitude of spontaneous activity in critical cognitive regions. These neuroimaging biomarkers may help identify patients at risk for cognitive decline and guide the development of targeted neuroprotective strategies during cancer treatment. The temporal dynamics of these changes underscore the importance of longitudinal monitoring and may inform optimal timing for cognitive interventions in cancer patients.

## Data Availability

The raw data supporting the conclusions of this article will be made available by the authors, without undue reservation.
